# Autologous peroneus longus tendon graft for superior fulcrum reconstruction: Maintained prospective 1-year outcomes at short-term final follow-up

**DOI:** 10.1371/journal.pone.0336413

**Published:** 2025-12-22

**Authors:** Kehao Wang, Cheng Peng, Lingchao Kong, Rende Ning, Zheng Zhu, Junke Wang, Guang Chen, Run Fang, Bizhi Tu

**Affiliations:** 1 The Third Affiliated Hospital of Anhui Medical University, Hefei, Anhui, China; 2 The Fifth Clinical Medical College of Anhui Medical University, Hefei, Anhui, China; 3 Hefei City’s First People’s Hospital, Hefei, Anhui, China; Menoufia University, EGYPT

## Abstract

**Background:**

Massive irreparable rotator cuff tears (MIRCTs) present challenges in terms of traditional treatments and can result in pain and functional impairment. Due to the limitations of traditional treatments for MIRCTs, alternative options such as superior fulcrum reconstruction (SFR) using the autologous peroneus longus tendon (PLT) have been explored. This study aims to evaluate the clinical and radiographic outcomes of SFR with autologous PLT grafts for the treatment of MIRCTs after a minimum follow-up period of 1 year.

**Methods:**

This was a prospective cohort study. Thirty-six patients with MIRCTs who underwent arthroscopic SFR with PLT grafts were enrolled and prospectively followed for a minimum of 1 year. Clinical and radiographic evaluations were performed preoperatively and at 3, 6, and 12 months postoperatively. Follow-up evaluations included assessments using the American Shoulder and Elbow Surgeons (ASES) score, Subjective Shoulder Value (SSV), visual analog scale (VAS), Quick-Disabilities of the Arm, Shoulder, and Hand (DASH) score, and measurements of shoulder joint range of motion. Radiography and MRI were used to evaluate the acromiohumeral distance (AHD), Hamada grade, and graft integrity. Repeated measures ANOVA was used to analyze the within-group and between-group differences under different conditions, followed by Bonferroni post-hoc tests to compare outcomes in the postoperative alignment subgroups.

**Results:**

At the 1-year assessment (n = 36), 34 patients (94.4%) healed well and 2 (5.6%) had MRI-confirmed graft failure. Significant improvements were observed in ASES, QuickDASH, SSV, VAS, forward flexion, external rotation, internal rotation, and AHD scores (all P < 0.05). The use of autologous PLT grafts in SFR resulted in favorable functional outcomes, with a high graft healing rate at the 1-year follow-up.

**Conclusions:**

Significant improvements were observed in the ASES, QuickDASH, SSV, VAS scores, and shoulder joint range of motion, highlighting the effectiveness of this approach for patients with MIRCTs.

## Introduction

Massive rotator cuff tear refers to a defect larger than 5 cm or that affects at least two rotator cuff tendons. When such a tear cannot be completely repaired due to tendon retraction, fat infiltration, or muscle atrophy, it is classified as a massive irreparable rotator cuff tear (MIRCT) [1]. MIRCTs are frequently associated with significant pain and result in functional impairment. This is primarily attributed to complications, including irreversible tendon retraction, fatty infiltration, and muscle atrophy, which pose significant challenges in treatment [2,3]. Traditional treatment approaches encounter various issues, such as a partial repair re-tear rate reaching as high as 60%–80% [4]. Consequently, reverse shoulder arthroplasty is typically reserved for older patients with reduced shoulder movement demand [5–7].

The introduction of the superior capsular reconstruction (SCR) procedure has garnered considerable interest as an approach for managing MIRCTs. This innovative technique employs either fascia lata autografts (FLAs) or human dermis autografts (HDAs) to reconstruct the superior capsule [8]. Its primary objective is to prevent the excessive upward movement of the humeral head during arm elevation, thereby mitigating pain and enhancing shoulder mobility. However, this approach has some limitations, including long-term creep of the FLA [9,10], uncertain healing rates with HDAs [11], and potential complications at the donor site [12,13].

**Fig 1:**
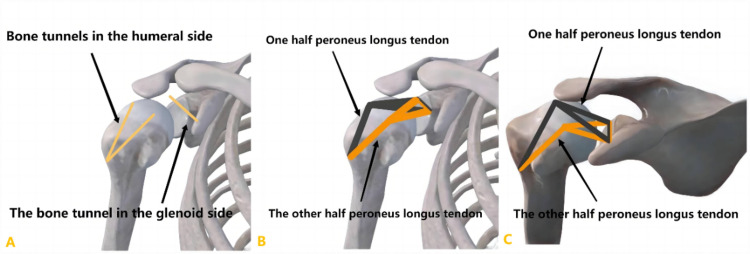
Surgical diagram. A, Bone tunnels; B, Schematic of tendon placement; C, Top view of the tendon placement schema.

Due to its good strength and sufficient size, the peroneus longus tendon (PLT) has been used as a reconstruction material for the cruciate ligament, medial patellofemoral ligament, lateral ankle ligament, and deltoid ligament [14–18]. PLTs have a favorable track record in ligament reconstruction and are known for their significant clinical efficacy, excellent tensile strength, and commendable tendon-to-bone healing rates [17]. In 2023, Ning et al introduced a surgical technique for superior fulcrum reconstruction (SFR) using an autologous PLT to treat MIRCTs[14]. Additionally, they employed an innovative surgical approach by replacing the traditional anchor method with a bone tunnel fixation method to create a mesh-like structure in the glenohumeral joint (Fig 1). This alteration enhances the interface area between the tendon and bone, thereby reducing the risk of fixation loosening. Given the potential advantages of this technique, our primary hypothesis was that, following a minimum 1-year follow-up, we could ensure the integrity of the graft and achieve satisfactory outcome. Therefore, this study aimed to comprehensively assess both the clinical and radiographic results in patients undergoing SFR.

## Materials and methods

This prospective cohort study enrolled patients from February 10, 2020 to June 27, 2023 with institutional review board approval. All participants provided written informed consent. Eligibility for unilateral SFR surgery was determined by sports medicine physicians using predetermined selection criteria. The analysis included only patients who: (1) met operative indications, (2) underwent successful SFR surgery, (3) demonstrated strict adherence to standardized postoperative rehabilitation protocols, and (4) completed scheduled follow-up assessments at 3 months, 6 months, and 1 year postoperatively. Written informed consent was obtained from all participants. The study protocol was approved by the Hefei First People’s Hospital Ethics Committee (Approval No. 2020-020-02). Signed consent documents are stored securely.

Patient selection was based on the presence of symptomatic MIRCTs, defined in this study as having ≥2 tendon tears (including the supraspinatus) or a tear of at least 5 cm in the supraspinatus, where the tear could not be completely repaired, and no observed functional impairments in the deltoid, latissimus dorsi, or pectoralis major muscles. Patients with a Hamada classification of 4 or 5, those who had received an intra-articular corticosteroid injection within the month before surgery, and those with glenohumeral arthritis, a labral injury, a history of a previous shoulder cuff repair surgery, pseudoparalysis, or a shoulder infection were excluded [19,20]. Repairable tears of the infraspinatus or subscapularis were all repaired during surgery; otherwise, patients were excluded from the study. The SFR procedure was only performed on symptomatic individuals with MIRCTs who met all inclusion criteria.

### Surgical technique

Each procedure was performed by an experienced surgeon. Patients were positioned in a beach chair setup under general anesthesia, and important anatomical points such as the clavicle, acromion, humeral head, and coracoid were marked with a marker. After standard sterilization and preparation, the arthroscopic tools were set up. The initial phase involved creating standard posterior viewing and anterior working portals, which were located just behind the acromion and in front of the coracoid. These portals allowed for arthroscopic inspections and internal assessments. After evaluating the extent of the damage, it was decided to repair both the infraspinatus and subscapularis muscles. The treatment plan for the biceps tendon—whether to sever, secure, or leave it untreated—was determined based on the patient’s age and the condition of the tendon. The arthroscope was then inserted into the subacromial area. To create a lateral anterior portal for operative access, a spinal needle was used as a guide. This portal was positioned approximately 3–4 cm from the acromion’s lateral boundary and aligned with the clavicle’s extension line. Additionally, a posterior lateral portal was created parallel to the anterior lateral portal, through which an arthroscope was inserted to examine the subacromial area. The synovial tissue within the subacromial space was then removed to expose the operation site, and standard subacromial decompression was performed. Subsequently, forceps were used to reduce torn rotator cuff tissue in order to assess the size and type of tear. Once an irreparable rotator cuff tear was confirmed, the decision was made to proceed with arthroscopic SFR surgery.

**Fig 2:**
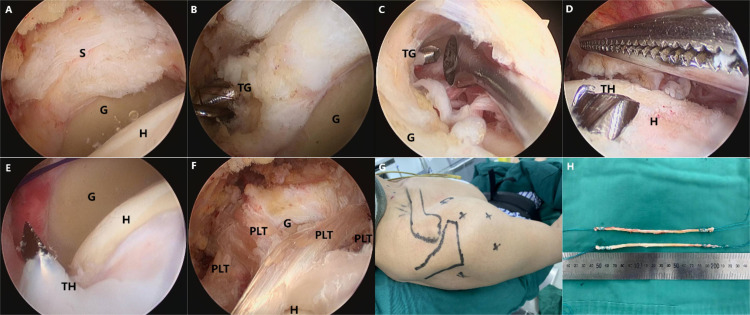
Superior fulcrum reconstruction with the peroneus longus tendon. An extensive irreparable rotator cuff tear was arthroscopically treated (A). A tunnel was drilled from the glenoid at 10:30 to the contralateral 1:30 position (B to C), and two bone tunnels were created in the humeral head (D to E). The graft was then passed through these tunnels, forming a suture bridge inside the glenohumeral joint (F). The patient is in the beach-chair position (G), and the peroneus longus tendon serves as the graft (H). (G, glenoid; TG, tunnel in glenoid side; H, humeral head; TH, tunnel in humeral head side; S, supraspinatus tendon; PLT, peroneus longus tendon as a graft).

To harvest the PLT, a 1-cm-long skin incision was made 3 cm proximal to the lateral malleolus on the affected side. The PLT insertion point was then identified and isolated. Using a tendon harvester, approximately half of the PLT was harvested from the distal end towards the proximal end and divided into two equal portions. The two tendon ends were then woven with non-absorbable polyester sutures of different colors at each end for differentiation. The woven tendon had a length of 14–16 cm and a diameter of 2–4 mm (Fig 2) and was stored in saline-moistened gauze.

A tunnel was drilled into the glenoid using a 4.5 mm drill bit through the posterior lateral portal. The tunnel’s entrance and exit were positioned at the 10:30 and 1:30 directions on the glenoid, respectively. During preparation, great care was taken to avoid damaging the bone cortex above and lateral to the tunnel. Two bone tunnels, each with a diameter of 4.5 mm, were created in the humeral head using a knee joint anterior cruciate ligament locator. The locator was inserted through the posterolateral portal, aligned at the point where the humeral head’s bone meets the cartilage, and then drilled towards the lateral and anterior aspects of the upper part of the humerus. The entry points of the two bone tunnels within the joint were separated by 2 cm and converged into a single exit on the lateral aspect of the upper humerus. On the glenoid side, a BI-type needle was used to thread the PDS suture (Ethicon) through the bone tunnel, while on the humeral head side, a grasper retrieved the suture’s end from the two tunnels. The two intertwined tendons were then combined and sequentially maneuvered through tunnels on the humeral head side, the glenoid side, and back through the humeral head side. The ends of these tendons were then extracted from the humeral side, and all four braided ends were positioned within the joint cavity.

The tendons were distinguished and separated; one tendon’s ends were guided through a bone tunnel on the humeral side using a grasper, and the other tendon’s ends followed through the adjacent tunnel. This configuration emulates an intra-articular suture bridge, which enhances joint stability. Subsequently, the ends of all threads were secured at the tunnel exits on the humeral side. After all surgical instruments were removed, the incision was closed, a sterile dressing was applied, and the area was bandaged to conclude the procedure.

### Rehabilitation

After the surgery, each patient’s shoulder was immobilized at 30° abduction using an abduction pillow (Table 1). Subsequently, active and passive range of motion exercises focused on the wrist and elbow joints were started within the first 2 weeks following the procedure. Isometric contraction exercises for the deltoid muscle were also initiated during this period. At six weeks post-surgery, the shoulder abduction brace was removed, and active-assisted motion exercises for the shoulder joint began. Additionally, throwing exercises were gradually introduced starting four months after the operation. Typically, normal activities could be resumed within 5–6 months after the surgery.

**Table 1:** Postoperative Rehabilitation Protocol.

Time Post-Surgery	Rehabilitation Measures
Immediately Post-op	Shoulder immobilized at 30° abduction using an abduction pillow
Within First 2 Weeks	Initiated active/passive ROM exercises for wrist and elbow Begun isometric deltoid contraction exercises
6 Weeks	Abduction brace removed Active-assisted shoulder ROM exercises initiated
4 Months	Gradually introduced throwing exercises
5-6 Months	Typically resumed normal activities

### Functional and radiological evaluation

Postoperative follow-up assessments were conducted at 3, 6, and 12 months to evaluate clinical and imaging outcomes before and 1 year after surgery. The primary outcome measure was the American Shoulder and Elbow Surgeons (ASES) index score [21]. Secondary outcomes included: Visual Analog Scale (VAS) pain scores, Subjective Shoulder Value (SSV) scores, Quick Disabilities of the Arm, Shoulder and Hand (Quick-DASH) scores, and shoulder range of motion measurements. For range of motion assessment, measurements were performed with the patient seated using a high-precision digital limb goniometer (Delixi Group Co., Ltd.). s [22]. Outcomes were considered satisfactory when achieving a final ASES score >50 points plus ≥17-point postoperative improvement without requiring SFR graft revision [23].

Standard shoulder radiographs included preoperative and postoperative upright anteroposterior views to measure the acromiohumeral distance (AHD) and apply the Hamada classification. Preoperative MRI was used to examine the size of supraspinatus tears, including tear presence and muscle atrophy, to assess the SFR graft’s integrity and size, along with the condition of the remaining rotator cuff muscles. At 6 months and 1 year post-surgery, MRI was used for qualitative and structural evaluations of the rotator cuff tendon and to assess the integrity of the repair. The presence of a full-thickness defect within the graft was diagnosed as a graft tear. In contrast, a graft without a full-thickness defect was diagnosed as a healed graft [24]. The severity of the rotator cuff injury and the level of fatty infiltration as determined by the modified Goutallier classification [25].

### Statistical analyses

Patients’ demographic and clinical data were presented as mean ± standard deviation (SD) for continuous variables and as frequencies (counts, n) for categorical variables. Group comparisons were performed using independent samples t-tests and chi-square tests, respectively. For functional and imaging indicators, all continuous variables were assessed for normality using Shapiro-Wilk tests prior to selecting parametric or nonparametric tests. Variables with *p > 0.05* for normality were analyzed using repeated-measures ANOVA; otherwise, the Friedman test with Dunn-Bonferroni post hoc correction was applied. Data homogeneity of variances was confirmed via Levene’s test. A one-way ANOVA was used for comparison between the infraspinatus repair group and the intact group.

## Results

### Demographic characteristics

**Fig 3:**
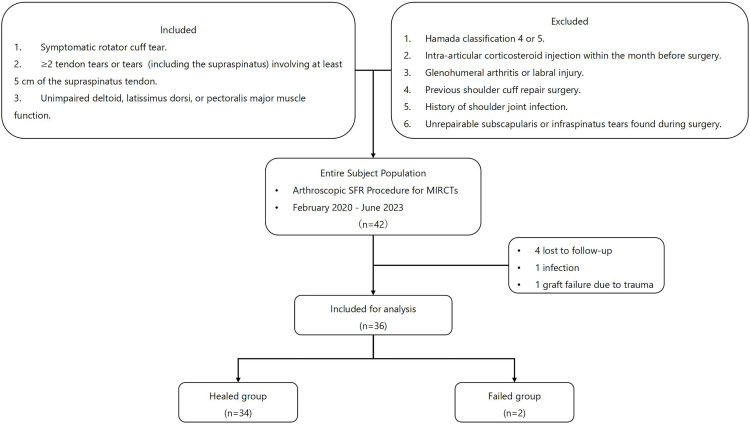
Research flow chart.

We assessed 42 patients who underwent SFR of the peroneus longus tendon. Sample size was determined through prospective power calculation and aligned with comparable arthroscopic studies [26–28]. Four patients who were unable to complete the follow-up period and two who underwent revision surgery due to infection and trauma were excluded from the study (Fig 3). All participants were followed for over 12 months, except for two cases where graft failure occurred at 4 and 11 months, leading to revision surgery. The mean follow-up was 12.8 months. The demographic data is presented in Table 2. Group comparisons for continuous and categorical variables were performed using the t-test and chi-square test, respectively. The analysis of demographic indicators revealed that for BMI, the p-value was 0.026 (< 0.05), suggesting a potentially significant difference between the groups. No statistically significant differences were found for the other variables.

**Table 2:** Patients’ demographic and clinical data.

Variable Data	Value	P
Age (years)	57.6 ± 8.5 (42–74)	0.09
Sex (n)	Male, 19; female, 15	0.31
Affected side (n)	Right, 24; Left, 10;	0.37
Dominant arm (n, D/ND)	D, 28; ND, 6	0.35
Body mass index, kg/m2	25.5 ± 2.8 (20.5–29.4)	0.03
Diabetes (n)	11	0.16
Smoking (n)	16	0.47
Hamada grade on radiographs	G1,19; G2, 14; G3, 3	
Rotator Cuff Tear Size (cm)	5.6 ± 0.5	
Long head of the biceps lesion (n)	25	0.55
Subscapularis lesion (n)	18	0.41
Infraspinatus lesion (n)	24	0.64

*D dominant, *ND* nondominant.

NOTE. The data are presented as numbers or mean ± SD (range).

### Functional outcomes

This study demonstrated significant enhancements in ASES function scores, SSV scores, QuickDASH scores, VAS pain scores, FE, ER, and IR at 1-year post-surgery assessments compared with pre-surgery values (Table 3). The primary outcome measure, the ASES score, significantly increased from 51.2 ± 16.1 points preoperatively to 79.9 ± 10.4 points at the 1-year follow-up (P < 0.001). At the 1-year follow-up, 32 patients (94%) achieved both the minimal clinically important difference (MCID) and the substantial clinical benefit (SCB) for the ASES score (defined as 17.5 points). Additionally, regardless of whether infraspinatus repair was performed, favorable outcomes were observed in the ASES score, VAS pain score, SSV score, and QuickDASH score at the one-year postoperative follow-up, as well as in FE, ER and IR. In one case, the patient developed shoulder adhesion 5 months after surgery; however, satisfactory recovery of shoulder function was achieved through guided functional exercises by a rehabilitation physician.

**Table 3:** Preoperative and postoperative subjective and objective outcomes.

	Preoperative	6 months postoperative	P-value	1 year postoperative	P-value
ASES	51.2 ± 16.1	70.5 ± 13.4	<0.001^a^	79.9 ± 10.4	<0.001^a^
VAS pain	6.7 ± 1.7	3.1 ± 1.4	<0.001^b^	2.0 ± 1.2	<0.001^b^
SSV	38.2 ± 17.1	65.3 ± 15.6	<0.001^a^	80.9 ± 14.2	<0.001^a^
QuickDASH	49.6 ± 17.1	29.1 ± 10.0	<0.001^a^	16.8 ± 7.9	<0.001^a^
Rom					
FE (°)	118.8 ± 18.6	132.8 ± 19.3	<0.001^a^	141.2 ± 17.5	<0.001^a^
ER (°)	44.3 ± 11.5	50.2 ± 12.6	<0.001^a^	55.5 ± 12.1	<0.001^a^
IR	11.2 ± 2.2	10.6 ± 2.2	<0.001^a^	10.2 ± 2.0	<0.001^a^

*ASES, American Shoulder and Elbow Surgeons; VAS, visual analog scale; SSV, subjective shoulder value; QuickDASH, Quick-Disabilities of the Arm, Shoulder, and Hand; ROM, range of motion; FE, forward elevation; ER, external rotation; and IR, internal rotation.

^a^ : Repeated measures ANOVA.

^b^ : Friedman Test.

### Radiologic results

**Fig 4:**
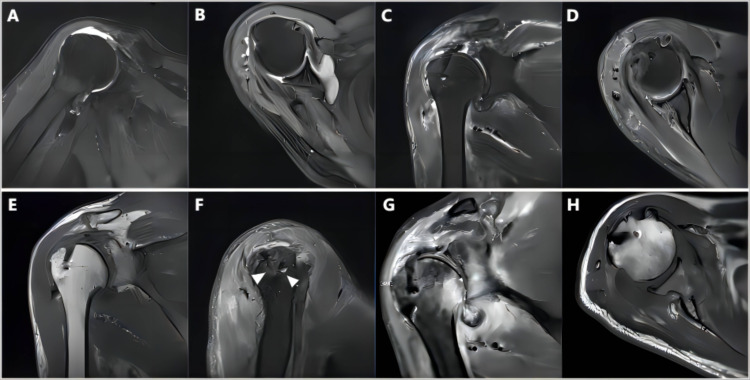
MRI images before and after surgery. A, B: Preoperative MRI images; C, D, E, F: MRI images 3 months post-surgery, where C shows the coronal plane in the T2 phase, D is a cross-sectional MRI, E represents the coronal plane in the T1 phase, and F displays the MRI in the sagittal plane with bone tunnels indicated by arrows; G, H: MRI images 6 months post-surgery.

Table 5 presents the X-ray and MRI results. According to the X-ray results, the preoperative AHD averaged 5.6 mm. At 6 months postoperatively, it increased to 7.7 mm, then decreased to 7 mm at 1 year postoperatively (P ≤ 0.01). Nineteen patients had a Hamada grade 1 classification before the operation. This increased to 30 patients at 6 months postoperatively and then decreased to 25 patients at 1 year postoperatively. MRI evaluation revealed that among the remaining 34 patients, two experienced graft failure at postoperative months 4 and 11, respectively. The MRI-assessed tendon healing rate was 94%. Both patients showed a graft tear at the side of the greater tuberosity of the humerus. All other patients did not experience any significant complications, such as graft re-tear (Fig 4).

**Table 5:** Comparison of preoperative and postoperative outcomes between groups with intact infraspinatus muscles and those with a repaired infraspinatus.

	infraspinatus intact	infraspinatus repair	*P*-value
ASES			
Preoperative	57.4 ± 16.9	48.7 ± 15.3	0.151
6 months	75.9 ± 13.4^	68.2 ± 13.0^	0.129
1 year	83.3 ± 10.6^	78.5 ± 10.2^	0.222
VAS			
Preoperative	5.8 ± 1.4	7.1 ± 1.7	0.047
6 months	2.8 ± 1.3^	3.3 ± 1.5^	0.365
1 year	1.7 ± 1.3^	2.2 ± 1.2^	0.306
SSV			
Preoperative	45.0 ± 17.2	35.4 ± 16.7	0.140
6 months	71.0 ± 15.2^	62.9 ± 15.5^	0.173
1 year	86.0 ± 15.8^	78.8 ± 13.3^	0.179
QuickDASH			
Preoperative	42.3 ± 17.6	52.7 ± 16.3	0.108
6 months	24.5 ± 10.7^	31.1 ± 9.3^	0.084
1 year	13.4 ± 7.0^	18.2 ± 8.0^	0.110
FE (°)			
Preoperative	126.9 ± 19.4	115.4 ± 17.6	0.101
6 months	142.6 ± 20.3^	128.8 ± 17.8^	0.096
1 year	152.6 ± 17.2^	136.4 ± 15.5^	0.064
ER (°)			
Preoperative	48.5 ± 11.3	42.5 ± 11.3	0.168
6 months	55.6 ± 11.9^	48.3 ± 11.7^	0.071
1 year	59.9 ± 12.1^	53.7 ± 11.8^	0.049
IR			
Preoperative	10.7 ± 2.4	11.4 ± 2.2	0.386
6 months	9.8 ± 2.2^	11.0 ± 2.1^	0.282
1 year	9.4 ± 2.2^	10.5 ± 1.9^	0.325

*ASES, American Shoulder and Elbow Surgeons; VAS, visual analog scale; SSV, subjective shoulder value, QuickDASH, Quick-Disabilities of the Arm, Shoulder, and Hand; FE, forward elevation; ER, external rotation; IR, internal rotation.

^P < 0.05.

## Discussion

This study demonstrates that arthroscopic SFR using autologous PLT grafts is safe and effective. In all 34 patients, the implanted PLT grafts remained intact with no donor-site complications observed, accompanied by significant improvements in range of motion. In contrast, Ohta et al.’s SCR study reported a 10.2% graft tear rate, 4.1% infection rate, and insufficient recovery of internal rotation strength in patients with concomitant subscapularis tendon tears [29]. Our results showed a consistent increase in ASES scores from preoperative (51.2) to 6 months (70.5) and 1 year postoperative (79.9). Similarly, Lim et al. [30] reported positive clinical outcomes with significant functional improvement and increased AHD (5.3 mm to 6.4 mm) in MIRCT patients treated with arthroscopic SCR; our study likewise observed AHD improvement from 5.6 mm preoperatively to 7.0 mm at one year postoperatively. Follow-up evaluations further confirmed the aforementioned improvements, which were evidenced by satisfactory graft healing, concomitant enhancement in functional mobility, and synchronous amelioration of imaging outcomes. However, Fried et al.’s 30-month SCR follow-up revealed only 50% return-to-work in manual laborers (60% to original positions), 47.6% sports resumption (33.3% achieving preoperative levels), a 14.8% reoperation rate, and 81.2% of non-returned workers still experiencing shoulder symptoms [31]. Ansgar Ilg et al.’s SCR study showed pain scores decreased from 4.2 to 1.0 with functional improvement at 2 years, but graft integrity was preserved in only 68.2% of cases [32], while Ferrando et al. [33] found a 25% graft failure rate in patients followed >2 years post-SCR. Additionally, animal studies by Peng et al. suggest that compared to SCR, SFR may yield superior outcomes in terms of collagen fiber maturity, fibrocartilage regeneration, and tendon regeneration [34]. Biomechanical investigations by Wang et al. further support the favorable performance of SFR [35,36]. Notably, although our results demonstrated statistically significant improvements, the clinical significance requires further validation through large-sample, multicenter studies.

The infraspinatus serves as a primary dynamic stabilizer of the shoulder joint. Multiple studies underscore the critical role of posterior rotator cuff integrity in glenohumeral function. Oh et al. [37] specifically demonstrated that repair of posterior rotator cuff tears (particularly the infraspinatus) is essential to restore normal glenohumeral kinematics in the setting of pathological kinematics.

Our study stratified patients into cohorts based on infraspinatus tear status to specifically evaluate the SFR technique’s clinical efficacy when managing shoulders with concomitant posterior cuff pathology. Crucially, the results demonstrate that SFR achieved comparable clinical outcomes irrespective of infraspinatus tear status, supporting its effectiveness in anatomically complex presentations (Table 4).

**Table 4:** Radiographic rotator cuff tear characteristics and outcomes.

	Preoperative	6 months postoperative	P-value	1 year postoperative	P-value
Acromiohumeral distance (mm)	5.6 ± 1.5	7.7 ± 1.5	<0.001^b^	7.0 ± 1.7	<0.001^b^
Hamada classification (*n*)	G1 (19)	G1 (30)	/	G1 (25)	/
Goutallier	1.9 ± 0.7	2.0 ± 0.8	0.467^b^	2.1 ± 0.8	0.146^b^
Sugaya	/	1.9 ± 0.7	/	2.3 ± 0.7	/
Graft integrity	/	Integrity 35	/	Integrity 34	/

*Goutallier staging has 4 stages (1–4), presented in the table as mean ± standard deviation. Sugaya grading comprises 5 stages (1–5), also shown in the table as mean ± standard deviation.

^b^ : Friedman Test.

In traditional SCR surgeries, HDAs and FLAs are used for surface coverage. The advantage is that the graft area can be large enough to cover the humeral head, but the drawback is that the strength of the graft is often insufficient, which may lead to issues such as graft creep. The use of FLA for SCR surgery introduces complications related to obtaining a sufficient amount of FLA from the donor site. Generally, a graft with a thickness ranging from 6 to 8 mm is required to achieve satisfactory clinical outcomes [38,39]. Achieving a minimum thickness of 6 mm often involves folding the FLA multiple times, especially if the initial graft is thin. This requires a substantial volume of FLA that includes muscle intervals within the graft, potentially leading to postoperative pain in the thigh and other complications. On the other hand, the use of HDA in SCR surgery has its own set of complications, such as a lack of mechanical strength [8] and high re-tear rates [40] when applied in a solitary layer. Notably, Nimura et al. [41] reported that the HDA used for SCR in their study was elongated by approximately 15% compared to FLA. In the present study, while the AHD increased by 1.4 mm at the 1-year follow-up compared to preoperative measurements—currently supporting its efficacy in the short term—a gradual decline of 0.7 mm was observed relative to the 6-month postoperative values. The long-term functional implications of this reduction remain unclear. However, this technique has gained increasing traction in clinical practice. Emerging biomechanical and histological evidence, particularly highlighted in recent 2025 studies, supports its biological rationale and demonstrates promising outcomes [34–36]. Despite this potential, the follow-up period remains relatively short. Confirming long-term efficacy and functional sustainability necessitates larger, multicenter studies with extended follow-up durations. We anticipate that further investigation will provide more robust evidence regarding its long-term benefits.

One significant advantage of using tendons for reconstruction is that, compared to the surface coverage provided by HDAs and FLAs, tendons offer sufficient strength. Currently, commonly used tendons include the long head of the biceps tendon and hamstring tendons. The “Chinese Way” technique for reconstructing the superior capsule using the long head of the biceps tendon [42] requires a high level of integrity and stability of the tendon itself. In this study, 27 out of 36z patients experienced long head of the biceps lesions, indicating that the biceps tendon is not always suitable as a graft material. Additionally, it may lead to the development of a Popeye sign and pain originating from the “biceps anchor” due to proximal biceps degeneration. Using autologous hamstring tendons [43] for superior capsule reconstruction also presents issues such as stress concentration at the glenoid anchor, a smaller tendon-to-bone healing area, significant trauma from open surgery, and potential graft wastage.

In our study, we utilized the validated PLT as a graft material due to its excellent strength, which far exceeds the tension required for effectively restoring the initial vector with the humeral head and scapula [44]. Additionally, its appropriate length and autograft characteristics, along with low harvesting costs and minimal complications, were also reasons for choosing the PLT.

Additionally, PLT, as an autograft, has a higher healing potential compared to allografts. Yildiz et al. [45] conducted a study comparing the effects of autograft tension band FLA and HDA on chronic supraspinatus tendon tears in rabbits. Their findings demonstrated that rabbits treated with autografts exhibited enhanced collagen fiber density, improved alignment of collagen fibers, and reduced presence of inflammatory cells, indicating effective tendon-to-bone healing. Furthermore, in our follow-up observations, we found no significant complications associated with the procurement of PLT in patients who underwent the procedure.

The force balance of the glenohumeral joint is crucial for its stability [46]. Specifically, in the coronal plane, the combined action of the deltoid and rotator cuffs is essential for the force balance and normal functioning of the shoulder [47,48]. These muscles help maintain the rotational center and equilibrium between the upper and lower forces of the rotator cuff. Rotator cuff tears disrupt this force couple, which is essential for maintaining a stable fulcrum for shoulder movements. This disruption leads to superior migration of the humeral head, resulting in shear forces and a significant reduction in both elevation and rotational functions [21,49]. Our technique aids in reconstructing the fulcrum. Unlike SCR, which provides active contraction force, our method utilizes “suture bridge-like fixation” using bone tunnels, offers strong initial fixation, and limits superior migration during shoulder movement. This approach effectively manages pain and improves function by preserving the force couple. Consequently, it restores the fulcrum and enhances range of motion compared to traditional SCR methods.

For MIRCTs, the surgical goal is to preserve the rotational center, prevent pseudoparalysis [50], eliminate pain, and help patients return to normal life. Our surgical approach aims to restore the upper and lower force balance of the rotator cuff and stabilize the humeral head during glenohumeral abduction. We achieve this by creating a mesh structure on the humeral head and applying significant downward pressure using the high-strength PLT. This helps limit the superior translation of the humeral head, increase the AHD, restore joint function, prevent humeral head impingement, and alleviate shoulder joint pain. Additionally, the reduced distance between the humeral head and acromioclavicular joint decreases impingement of the graft tendon with the humeral head, reduces wear on the graft tendon, and prolongs its lifespan. In the transverse plane, the force balance is maintained by the anterior (subscapularis) and posterior (infraspinatus and teres minor) parts of the rotator cuff [51]. Therefore, we repaired the tears in the subscapularis and infraspinatus muscles during surgery.

Moreover, this surgical procedure is simpler and more economically advantageous than traditional SCR surgery. In addition to using autografts, it requires minimal consumables, such as anchor screws. Furthermore, unlike the balloon spacer, which tends to degrade over time [52,53], the peroneus longus tendon autograft offers a durable and enduring solution for the glenohumeral joint, eliminating concerns about biodegradation.

In this study, the graft was secured using bone tunnels, a fixation method that has shown favorable outcomes in animal experiments and biomechanical studies [54–56]. Bone tunnels provide an increased tendon-bone contact area, offering more growth bases for tendon-to-bone healing. Zeng et al. [57] demonstrated that the bone tunnel technique achieved superior tendon-bone healing for rotator cuff tears in a rabbit model compared to the conventional on-surface repair method. Zhao [58] used the Arthroscopic “Inlay” Bristow Procedure with Suture Button Fixation for the treatment of recurrent anterior glenohumeral instability, achieving good results by increasing the bone healing area to promote bone union. In reverse shoulder arthroplasty, the inlay fixation method has achieved good clinical outcomes and may also help improve long-term results [59,60]. Inlay biceps tenodesis for long head of biceps tendinopathy can also results in improved clinical outcomes [61]. Inspired by the favorable outcomes achieved with the inlay technique, this surgical approach adopts the inlay method, moving away from the traditional onlay approach of securing the graft onto the bone surface with anchors. Instead, it uses the tendon-bone tunnel (inlay) fixation technique, which promotes improved tendon-bone healing.

Additionally, this structure, designed to mimic a tension band [62], leverages the principles of tension to support early functional exercises. By applying controlled stress through the PLT on the bone tunnel during initial movements, this approach promotes tendon-to-bone healing while allowing functional exercises, ultimately contributing to improved long-term outcomes. Similarly, in SCR, four anchors secure the graft at both the glenoid and humeral head, resembling the double-row repair approach. By employing bone tunnels, our repair method aligns with the principles of the suture bridge method, which is known for its enhanced biomechanical characteristics [63,64].

In the present study, both graft failures occurred on the humeral side. One case involved a 72-year-old male with a BMI of 26.9, a dominant-side rotator cuff injury, diabetes, and a smoking habit; the graft failed at 11 months. The other case involved a 67-year-old male with diabetes and a dominant-side injury, whose graft failed 4 months postoperatively. Consistent with previous studies by our team and Lee et al. [65], humeral-side detachment remains the most frequent failure mode, with up to 80% of tears occurring at this site. This may be partly attributed to excessive tensile and shear stress concentrated at the lateral fixation point during active shoulder elevation, as the graft functions as a fulcrum over a larger motion arc relative to the glenoid side.

From a biomechanical perspective, the graft may not maintain full isometry throughout the shoulder’s range of motion. Non-isometric elongation patterns may generate uneven loading, and at specific joint angles, increased tensile force may amplify stress concentration at the humeral anchor site. In patients with impaired biological healing capacity, such as those with diabetes, this mechanically unfavorable environment may predispose the graft–bone interface to early failure.

Furthermore, epidemiological and clinical studies have demonstrated that diabetes significantly alters tendon structure and functional properties, increasing the risk of tendinopathy and rupture by over threefold compared to non-diabetic individuals [66]. Hyperglycemia-induced microvascular dysfunction triggers collagen disorganization, aberrant angiogenesis, and inflammation, ultimately impairing tendon–bone integration [67,68]. In this study, both early humeral-side failures occurred in diabetic patients, suggesting that hyperglycemia-mediated endothelial injury and reduced healing potential may have exacerbated the vulnerability of the fixation site under non-isometric stress.

Additionally, MIRCTs frequently occur in elderly patients, in whom osteoporosis is commonly present. Low bone mineral density in the greater tuberosity compromises initial anchor fixation strength and delays osseointegration. When combined with the non-isometric loading pattern and diabetes-related healing impairment, osteoporosis may further increase the risk of graft loosening or rupture.

Notably, among the 34 patients with satisfactory healing, 11 had comorbid diabetes; meanwhile, both failure cases occurred in diabetic individuals. Although this suggests a possible association, the current sample size is insufficient to confirm diabetes as an independent risk factor. Further studies incorporating biomechanical loading analysis and stratification by metabolic and bone quality status are warranted to clarify the interplay among diabetes, graft mechanics, and osteoporosis.

Looking ahead, further studies are warranted to stratify patients based on metabolic (e.g., diabetes) and skeletal (e.g., osteoporosis) conditions to identify specific clinical risk factors for graft failure. Subgroup analyses may clarify whether these comorbidities act independently or synergistically to compromise tendon–bone healing. In addition, finite element analysis could be utilized to simulate graft tension patterns across different shoulder motion angles and variable bone quality conditions. Such biomechanical modeling would help elucidate how non-isometric loading interacts with impaired biological healing capacity to influence fixation outcomes. These investigations may provide a more comprehensive mechanistic basis for optimizing surgical strategies and tailoring postoperative management in high-risk populations.

This study has the following limitations: All surgeries were performed by a single shoulder surgeon, potentially introducing single-surgeon bias that may influence outcomes due to operator-dependent factors, thereby limiting the generalizability of the findings; the patient cohort was relatively small and lacked a control group (e.g., patients undergoing arthroscopic superior capsule reconstruction), with subgroup analyses revealing underpowered subgroups; four cases lost to follow-up may have introduced selection bias; although no significant peroneus longus tendon donor-site complications or neurologic symptoms were observed, the absence of a structured assessment protocol necessitates further investigation to confirm long-term safety; additionally, the impact of graft thickness and rotator cuff tear extent on clinical outcomes was not explored, and inter-observer agreement for imaging assessments (e.g., tendon healing) remains unvalidated. Finally, despite favorable short-term outcomes, future multicenter, large-scale studies with long-term follow-up are warranted.

## Conclusion

This study demonstrates that arthroscopic SFR achieved significant clinical efficacy at 1-year follow-up. The technique, supplemented with autologous peroneus longus tendon grafting, yielded high graft integration rates alongside statistically significant improvements in functional scores (ASES, SSV, QuickDASH), pain indices (VAS), and shoulder range of motion. Collectively, these outcomes validate the clinical utility of the SFR approach for managing MIRCTs.

## References

[1] Li H, Zhou B, Tang K. Advancement in Arthroscopic Superior Capsular Reconstruction for Irreparable Massive Rotator Cuff Tear. Orthop Surg. 2021;13(7):1951–9. doi: 10.1111/os.12976 34585538 PMC8528972

[2] Gerber C, Wirth SH, Farshad M. Treatment options for massive rotator cuff tears. J Shoulder Elbow Surg. 2011;20(2 Suppl):S20–9. doi: 10.1016/j.jse.2010.11.028 21281919

[3] Ditsios K, Boutsiadis A, Kapoukranidou D, Chatzisotiriou A, Kalpidis I, Albani M, et al. Chronic massive rotator cuff tear in rats: in vivo evaluation of muscle force and three-dimensional histologic analysis. Journal of shoulder and elbow surgery. 2014;23(12):1822–30. doi: 10.1016/j.jse.2014.04.016 24981552

[4] Prabhakar A, Kanthalu Subramanian JN, Swathikaa P, Kumareswaran SI, Subramanian KN. Current concepts on management of cuff tear. J Clin Orthop Trauma. 2022;28:101808. doi: 10.1016/j.jcot.2022.101808 35402155 PMC8983388

[5] Kobayashi EF, Oak SR, Miller BS, Bedi A. Treatment of Massive Rotator Cuff Tears with Reverse Shoulder Arthroplasty. Clin Sports Med. 2023;42(1):157–73. doi: 10.1016/j.csm.2022.08.007 36375867

[6] Ek ETH, Neukom L, Catanzaro S, Gerber C. Reverse total shoulder arthroplasty for massive irreparable rotator cuff tears in patients younger than 65 years old: results after five to fifteen years. J Shoulder Elbow Surg. 2013;22(9):1199–208. doi: 10.1016/j.jse.2012.11.016 23385083

[7] Ammitzboell M, Baram A, Brorson S, Olsen BS, Rasmussen JV. Poor patient-reported outcome after shoulder replacement in young patients with cuff-tear arthropathy: a matched-pair analysis from the Danish Shoulder Arthroplasty Registry. Acta Orthop. 2019;90(2):119–22. doi: 10.1080/17453674.2018.1563855 30669910 PMC6461079

[8] Mihata T, Bui CNH, Akeda M, Cavagnaro MA, Kuenzler M, Peterson AB, et al. A biomechanical cadaveric study comparing superior capsule reconstruction using fascia lata allograft with human dermal allograft for irreparable rotator cuff tear. J Shoulder Elbow Surg. 2017;26(12):2158–66. doi: 10.1016/j.jse.2017.07.019 29146012

[9] Bi M, Ding W, Zheng M, Peng Z, Li J, Ding S. Arthroscopic Superior Capsule Reconstruction With Combined Fascia Lata Autograft and Synthetic Scaffold Patch Graft for the Treatment of Irreparable Rotator Cuff Tears Yields Favorable Clinical and Radiographic Outcomes at Minimum 2-Year Follow-Up. Arthroscopy. 2023;39(8):1800–10. doi: 10.1016/j.arthro.2023.02.025 36924836

[10] Ding S, Ge Y, Zheng M, Ding W, Jin W, Li J, et al. Arthroscopic Superior Capsular Reconstruction Using “Sandwich” Patch Technique for Irreparable Rotator Cuff Tears. Arthrosc Tech. 2019;8(9):e953–9. doi: 10.1016/j.eats.2019.05.004 31687326 PMC6819740

[11] Denard PJ, Brady PC, Adams CR, Tokish JM, Burkhart SS. Preliminary Results of Arthroscopic Superior Capsule Reconstruction with Dermal Allograft. Arthroscopy. 2018;34(1):93–9. doi: 10.1016/j.arthro.2017.08.265 29146165

[12] de Campos Azevedo CI, Ângelo ACLPG, Vinga S. Arthroscopic Superior Capsular Reconstruction With a Minimally Invasive Harvested Fascia Lata Autograft Produces Good Clinical Results. Orthop J Sports Med. 2018;6(11):2325967118808242. doi: 10.1177/2325967118808242 30505873 PMC6259077

[13] Hirahara AM, Adams CR. Arthroscopic Superior Capsular Reconstruction for Treatment of Massive Irreparable Rotator Cuff Tears. Arthrosc Tech. 2015;4(6):e637-41. doi: 10.1016/j.eats.2015.07.006 26870638 PMC4738239

[14] Zhang Q, Ying L, Han D, Ye L, Tung T-H, Liang J, et al. Arthroscopic reconstruction of the medial patellofemoral ligament in skeletally immature patients using the modified sling procedure: a novel technique for MPFL reconstruction. J Orthop Surg Res. 2023;18(1):334. doi: 10.1186/s13018-023-03775-9 37147697 PMC10163800

[15] Kim HN, Dong Q, Hong DY, Yoon YH, Park YW. Percutaneous lateral ankle ligament reconstruction using a split peroneus longus tendon free graft: technical tip. Foot Ankle Int. 2014;35(10):1082–6. doi: 10.1177/1071100714540892 25015391

[16] Lui TH. Technical tips: reconstruction of deep and superficial deltoid ligaments by peroneus longus tendon in stage 4 posterior tibial tendon dysfunction. Foot Ankle Surg. 2014;20(4):295–7. doi: 10.1016/j.fas.2014.04.006 25457670

[17] Shi F-D, Hess DE, Zuo J-Z, Liu S-J, Wang X-C, Zhang Y, et al. Peroneus Longus Tendon Autograft is a Safe and Effective Alternative for Anterior Cruciate Ligament Reconstruction. J Knee Surg. 2019;32(8):804–11. doi: 10.1055/s-0038-1669951 30206913

[18] Punnoose DJ, Varghese J, Theruvil B, Thomas AB. Peroneus Longus Tendon Autografts have Better Graft Diameter, Less Morbidity, and Enhanced Muscle Recuperation than Hamstring Tendon in ACL Reconstruction. Indian J Orthop. 2024;58(7):979–86. doi: 10.1007/s43465-024-01185-5 38948366 PMC11208339

[19] Pashuck TD, Hirahara AM, Cook JL, Cook CR, Andersen WJ, Smith MJ. Superior Capsular Reconstruction Using Dermal Allograft Is a Safe and Effective Treatment for Massive Irreparable Rotator Cuff Tears: 2-Year Clinical Outcomes. Arthroscopy. 2021;37(2):489–96.e1. doi: 10.1016/j.arthro.2020.10.014 33080333

[20] Lacheta L, Horan MP, Schairer WW, Goldenberg BT, Dornan GJ, Pogorzelski J, et al. Clinical and Imaging Outcomes After Arthroscopic Superior Capsule Reconstruction With Human Dermal Allograft for Irreparable Posterosuperior Rotator Cuff Tears: A Minimum 2-Year Follow-Up. Arthroscopy. 2020;36(4):1011–9. doi: 10.1016/j.arthro.2019.12.024 31953193

[21] Preuss FR, Day HK, Peebles AM, Mologne MS, Provencher MT. Reverse Total Shoulder Arthroplasty for Treatment of Massive, Irreparable Rotator Cuff Tear. Arthrosc Tech. 2022;11(6):e1133–9. doi: 10.1016/j.eats.2022.02.022 35782844 PMC9244853

[22] Oh JH, Kim SH, Shin SH, Chung SW, Kim JY, Kim SH, et al. Outcome of rotator cuff repair in large-to-massive tear with pseudoparalysis: a comparative study with propensity score matching. Am J Sports Med. 2011;39(7):1413–20. doi: 10.1177/0363546511399865 21460068

[23] Cvetanovich GL, Gowd AK, Liu JN, Nwachukwu BU, Cabarcas BC, Cole BJ, et al. Establishing clinically significant outcome after arthroscopic rotator cuff repair. J Shoulder Elbow Surg. 2019;28(5):939–48. doi: 10.1016/j.jse.2018.10.013 30685283

[24] Hasegawa A, Mihata T, Fukunishi K, Itami Y, Uchida A, Neo M. Structural and clinical outcomes after superior capsule reconstruction using an at least 6-mm-thick fascia lata autograft including the intermuscular septum. J Shoulder Elbow Surg. 2023;32(2):e48–59. doi: 10.1016/j.jse.2022.07.010 35998778

[25] Fuchs B, Weishaupt D, Zanetti M, Hodler J, Gerber C. Fatty degeneration of the muscles of the rotator cuff: assessment by computed tomography versus magnetic resonance imaging. J Shoulder Elbow Surg. 1999;8(6):599–605. doi: 10.1016/s1058-2746(99)90097-6 10633896

[26] Ben H, Kholinne E, Guo J, Park JY, Jeon I-H. Combined Superior Capsular Reconstruction Using Fascia Lata Autograft and Lower Trapezius Transfer Using Achilles Tendon Allograft Are Associated With Improved Surgical Outcomes in Patients With Chronic Posterosuperior Irreparable Massive Rotator Cuff Tears. Arthroscopy. 2025;41(7):2248–58. doi: 10.1016/j.arthro.2024.11.096 39672244

[27] Lee KW, Choi HG, Yang DS, Yu YT, Kim WS, Choy WS. Achilles Tendon Allograft for Superior Capsule Reconstruction in Irreparable Massive Rotator Cuff Tears. Clin Orthop Surg. 2021;13(3):395–405. doi: 10.4055/cios20284 34484633 PMC8380524

[28] Joo M-S, Lee S-H, Kim D-K, Cho Y-H, Kim J-W. Outcomes After Superior Capsular Reconstruction With an Achilles Tendon-Bone Allograft Using the Modified Keyhole Technique: A 2-Year Follow-up of a Novel Technique for Irreparable Rotator Cuff Tears. Orthop J Sports Med. 2023;11(7):23259671231182327. doi: 10.1177/23259671231182327 37435426 PMC10331213

[29] Ohta S, Ueda Y, Komai O. Postoperative results of arthroscopic superior capsule reconstruction using fascia lata: a retrospective cohort study. J Shoulder Elbow Surg. 2024;33(3):686–97. doi: 10.1016/j.jse.2023.07.021 37619926

[30] Lim S, AlRamadhan H, Kwak J-M, Hong H, Jeon I-H. Graft tears after arthroscopic superior capsule reconstruction (ASCR): pattern of failure and its correlation with clinical outcome. Arch Orthop Trauma Surg. 2019;139(2):231–9. doi: 10.1007/s00402-018-3025-7 30167857

[31] Fried JW, Hurley ET, Colasanti CA, Lin CC, Jazrawi LM, Meislin RJ. Return to Work and Recreational Sport After Superior Capsule Reconstruction with Dermal Allograft. Bull Hosp Jt Dis (2013). 2023;81(3):168–72. 37639344

[32] Ilg A, Kaiser R, Schneider S, Hollander K, Holz J. Patient-Reported Outcomes After Arthroscopic Superior Capsule Reconstruction With an Acellular Porcine Dermal Xenograft for Irreparable Rotator Cuff Tears. Orthop J Sports Med. 2024;12(10):23259671241264499. doi: 10.1177/23259671241264499 39492874 PMC11529357

[33] Ferrando A, Kingston R, Delaney RA. Superior capsular reconstruction using a porcine dermal xenograft for irreparable rotator cuff tears: outcomes at minimum two-year follow-up. J Shoulder Elbow Surg. 2021;30(5):1053–9. doi: 10.1016/j.jse.2020.08.020 32890682

[34] Peng C, Li H, Wang K, Chen G, Kong L, Ning R. Superior fulcrum reconstruction improve tendon-to-bone healing in irreparable massive rotator cuff tears compared with superior capsule reconstruction. Sci Rep. 2025;15(1):21285. doi: 10.1038/s41598-025-09329-9 40595372 PMC12215457

[35] Wang K, Fang R, Chen G, Tu B, Wu H, Li H, et al. Superior Fulcrum Reconstruction for Massive Irreparable Rotator Cuff Tears Using the Peroneus Longus Tendon in 2 Variations: A Cadaveric Static Biomechanical Study. Orthop J Sports Med. 2025;13(7):23259671251349686. doi: 10.1177/23259671251349686 40626141 PMC12231963

[36] Wang K, Ning R, Fang R, Chen G, Li H, Peng C. Modified superior capsule reconstruction using the peroneus longus for irreparable massive rotator-cuff tears: A cadaveric study. J Back Musculoskelet Rehabil. 2025;38(2):352–63. doi: 10.1177/10538127241296766 39973267

[37] Oh JH, McGarry MH, Jun BJ, Gupta A, Chung KC, Hwang J, et al. Restoration of shoulder biomechanics according to degree of repair completion in a cadaveric model of massive rotator cuff tear: importance of margin convergence and posterior cuff fixation. Am J Sports Med. 2012;40(11):2448–53. doi: 10.1177/0363546512458775 22984129

[38] Mihata T, Lee TQ, Watanabe C, Fukunishi K, Ohue M, Tsujimura T, et al. Clinical results of arthroscopic superior capsule reconstruction for irreparable rotator cuff tears. Arthroscopy. 2013;29(3):459–70. doi: 10.1016/j.arthro.2012.10.022 23369443

[39] Scheiderer B, Kia C, Obopilwe E, Johnson JD, Cote MP, Imhoff FB, et al. Biomechanical Effect of Superior Capsule Reconstruction Using a 3-mm and 6-mm Thick Acellular Dermal Allograft in a Dynamic Shoulder Model. Arthroscopy: the journal of arthroscopic & related surgery: official publication of the Arthroscopy Association of North America and the International Arthroscopy Association. 2020;36(2):355–64. doi: 10.1016/j.arthro.2019.08.026 31791890

[40] Sommer MC, Wagner E, Zhu S, McRae S, MacDonald PB, Ogborn D, et al. Complications of Superior Capsule Reconstruction for the Treatment of Functionally Irreparable Rotator Cuff Tears: A Systematic Review. Arthroscopy. 2021;37(9):2960–72. doi: 10.1016/j.arthro.2021.03.076 33887411

[41] Nimura A, Kato A, Yamaguchi K, Mochizuki T, Okawa A, Sugaya H, et al. The superior capsule of the shoulder joint complements the insertion of the rotator cuff. J Shoulder Elbow Surg. 2012;21(7):867–72. doi: 10.1016/j.jse.2011.04.034 21816631

[42] Boutsiadis A, Chen S, Jiang C, Lenoir H, Delsol P, Barth J. Long Head of the Biceps as a Suitable Available Local Tissue Autograft for Superior Capsular Reconstruction: “The Chinese Way”. Arthrosc Tech. 2017;6(5):e1559–66. doi: 10.1016/j.eats.2017.06.030 29354474 PMC5709836

[43] Rosales-Varo AP, Zafra M, García-Espona MA, Flores-Ruiz MA, Roda O. Superior capsular reconstruction of irreparable rotator cuff tear using autologous hamstring graft. Rev Esp Cir Ortop Traumatol (Engl Ed). 2019;63(1):1–6. doi: 10.1016/j.recot.2018.08.004 30522961

[44] Omid R, Heckmann N, Wang L, McGarry MH, Vangsness CT Jr, Lee TQ. Biomechanical comparison between the trapezius transfer and latissimus transfer for irreparable posterosuperior rotator cuff tears. J Shoulder Elbow Surg. 2015;24(10):1635–43. doi: 10.1016/j.jse.2015.02.008 25847516

[45] Yildiz F, Bilsel K, Pulatkan A, Kapicioglu M, Uzer G, Çetindamar T, et al. Comparison of two different superior capsule reconstruction methods in the treatment of chronic irreparable rotator cuff tears: a biomechanical and histologic study in rabbit models. J Shoulder Elbow Surg. 2019;28(3):530–8. doi: 10.1016/j.jse.2018.08.022 30466819

[46] Zafra M, Carpintero P, Carrasco C. Latissimus dorsi transfer for the treatment of massive tears of the rotator cuff. Int Orthop. 2009;33(2):457–62. doi: 10.1007/s00264-008-0536-9 18392621 PMC2899072

[47] Nottage WM. Editorial Commentary: Partial (Shoulder Rotator) Cuff Repair: May the Force (Couple) Be With You. Arthroscopy. 2017;33(11):1956–7. doi: 10.1016/j.arthro.2017.08.237 29102010

[48] Inman VT, Saunders JB, Abbott LC. Observations of the function of the shoulder joint. 1944. Clin Orthop Relat Res. 1996;(330):3–12. doi: 10.1097/00003086-199609000-00002 8804269

[49] Adams CR, Pasqualini I, Heidenthal JM, Brady PC, Denard PJ. A Technique for a Suture-Based Cable Reconstruction of an Irreparable Posterosuperior Rotator Cuff Tear. Arthrosc Tech. 2022;11(11):e2055–60. doi: 10.1016/j.eats.2022.08.003 36457380 PMC9705767

[50] Mihata T, McGarry MH, Kahn T, Goldberg I, Neo M, Lee TQ. Biomechanical Effect of Thickness and Tension of Fascia Lata Graft on Glenohumeral Stability for Superior Capsule Reconstruction in Irreparable Supraspinatus Tears. Arthroscopy. 2016;32(3):418–26. doi: 10.1016/j.arthro.2015.08.024 26524937

[51] Burkhart SS. Arthroscopic treatment of massive rotator cuff tears. Clinical results and biomechanical rationale. Clin Orthop Relat Res. 1991;(267):45–56. doi: 10.1097/00003086-199106000-00006 2044292

[52] Singh S, Reeves J, Langohr GDG, Johnson JA, Athwal GS. The Subacromial Balloon Spacer Versus Superior Capsular Reconstruction in the Treatment of Irreparable Rotator Cuff Tears: A Biomechanical Assessment. Arthroscopy. 2019;35(2):382–9. doi: 10.1016/j.arthro.2018.09.016 30522801

[53] Stewart RK, Kaplin L, Parada SA, Graves BR, Verma NN, Waterman BR. Outcomes of Subacromial Balloon Spacer Implantation for Massive and Irreparable Rotator Cuff Tears: A Systematic Review. Orthop J Sports Med. 2019;7(10):2325967119875717. doi: 10.1177/2325967119875717 31663007 PMC6794659

[54] Mehta V, Mandala C, Akhter A. Cyclic Testing of 3 Medial Patellofemoral Ligament Reconstruction Techniques. Orthop J Sports Med. 2017;5(6):2325967117712685. doi: 10.1177/2325967117712685 28680899 PMC5490843

[55] Korth K, Bolam S, Leiferman E, Crenshaw T, Dray M, Crawford HA, et al. Histological and radiographic evaluation of three common tendon transfer techniques in an un-ossified bone porcine model: implications for early anterior tibialis tendon transfers in children with clubfeet. J Child Orthop. 2021;15(5):443–50. doi: 10.1302/1863-2548.15.210076 34858530 PMC8582610

[56] Zhao F, Hu X, Zhang J, Shi W, Ren B, Huang H, et al. A more flattened bone tunnel has a positive effect on tendon-bone healing in the early period after ACL reconstruction. Knee Surg Sports Traumatol Arthrosc. 2019;27(11):3543–51. doi: 10.1007/s00167-019-05420-7 30877317

[57] Zeng S, Sun J, Qin B, Liu Y, Liu G, Deng K, et al. Semi-Bone Tunnel Technique Using Double-Row Suture Bridge Combined With Platelet-Rich Plasma Hydrogel for Rotator Cuff Repair in a Rabbit Model. Am J Sports Med. 2024;52(5):1308–18. doi: 10.1177/03635465241235146 38523475

[58] Shao Z, Song Q, Cheng X, Luo H, Lin L, Zhao Y, et al. An Arthroscopic “Inlay” Bristow Procedure With Suture Button Fixation for the Treatment of Recurrent Anterior Glenohumeral Instability: 3-Year Follow-up. Am J Sports Med. 2020;48(11):2638–49. doi: 10.1177/0363546520943633 32813567

[59] Zitnay JL, Tashjian RZ, Walch G, Chalmers PN, Joyce CD, Henninger HB. Inlay vs. onlay humeral components in reverse total shoulder arthroplasty: a biorobotic shoulder simulator study. J Shoulder Elbow Surg. 2024;33(6):1377–86. doi: 10.1016/j.jse.2023.10.015 38036254 PMC11098709

[60] Lee H-J, Yoon C-Y, Kim Y-S. Comparison of Clinical Performance of Inlay versus Onlay Humerus Implants in Reverse Total Shoulder Arthroplasty. Clin Orthop Surg. 2023;15(1):135–44. doi: 10.4055/cios22084 36778983 PMC9880513

[61] Jackson GR, Meade J, Coombes K, Young BL, Hamid N, Piasecki DP, et al. Onlay Versus Inlay Biceps Tenodesis for Long Head of Biceps Tendinopathy: A Systematic Review and Meta-analysis. J Am Acad Orthop Surg Glob Res Rev. 2022;6(12):e22.00255. doi: 10.5435/JAAOSGlobal-D-22-00255 36732300 PMC9746747

[62] Bel J-C, Lefèvre C. Reconstruction of patella fractures with the tension band technique: A review on clinical results and tips and tricks. Injury. 2024;55 Suppl 1:111401. doi: 10.1016/j.injury.2024.111401 39069346

[63] Park MC, Tibone JE, ElAttrache NS, Ahmad CS, Jun B-J, Lee TQ. Part II: Biomechanical assessment for a footprint-restoring transosseous-equivalent rotator cuff repair technique compared with a double-row repair technique. J Shoulder Elbow Surg. 2007;16(4):469–76. doi: 10.1016/j.jse.2006.09.011 17321158

[64] Park MC, Idjadi JA, Elattrache NS, Tibone JE, McGarry MH, Lee TQ. The effect of dynamic external rotation comparing 2 footprint-restoring rotator cuff repair techniques. Am J Sports Med. 2008;36(5):893–900. doi: 10.1177/0363546507313092 18272799

[65] Lee S-J, Min Y-K. Can inadequate acromiohumeral distance improvement and poor posterior remnant tissue be the predictive factors of re-tear? Preliminary outcomes of arthroscopic superior capsular reconstruction. Knee Surg Sports Traumatol Arthrosc. 2018;26(7):2205–13. doi: 10.1007/s00167-018-4912-8 29594325

[66] Leong HT, Fu SC, He X, Oh JH, Yamamoto N, Hang S. Risk factors for rotator cuff tendinopathy: A systematic review and meta-analysis. J Rehabil Med. 2019;51(9):627–37. doi: 10.2340/16501977-2598 31489438

[67] Lui PPY. Tendinopathy in diabetes mellitus patients-Epidemiology, pathogenesis, and management. Scand J Med Sci Sports. 2017;27(8):776–87. doi: 10.1111/sms.12824 28106286

[68] Sayegh ET, Gooden MJ, Lowenstein NA, Collins JE, Matzkin EG. Patients with diabetes mellitus experience poorer outcomes after arthroscopic rotator cuff repair. JSES Int. 2021;6(1):91–6. doi: 10.1016/j.jseint.2021.08.007 35141681 PMC8811388

